# Understanding perceptions of ophthalmology residents on pursuing a uveitis fellowship

**DOI:** 10.3389/fopht.2026.1824816

**Published:** 2026-04-16

**Authors:** Lorenzo Bosque, John Gonzales, Shilpa Kodati, Meghan Berkenstock

**Affiliations:** 1 College of Medicine, Drexel University, Philadelphia, PA, United States; 2 Department of Ophthalmology, University of California San Francisco, San Francisco, CA, United States; 3 University of Michigan Department of Ophthalmology and Visual Sciences, Ann Arbor, MI, United States; 4 Ocular Immunology Division, The Johns Hopkins Wilmer Eye Institute, Baltimore, MD, United States

**Keywords:** ocular immunology, ophthalmology education, ophthalmology fellowship, ophthalmology residency, uveitis

## Abstract

**Purpose:**

To investigate factors influencing the decision of ophthalmology residents in the United States to pursue a uveitis fellowship.

**Methods:**

A cross-sectional survey, with prospective data collection, study. From December 2023 to July 2024, an anonymous survey was distributed via the Association of University Professors of Ophthalmology (AUPO) to all United States ophthalmology residents. The survey was distributed a second time, between May 2024 and July 2024, through the American Uveitis Society listserv to distribute to residents. The primary outcome was to determine factors influencing ophthalmology residents’ decisions to pursue or forgo a uveitis fellowship.

**Results:**

Of 115 ophthalmology resident respondents, 14 (12.2%) chose to pursue a uveitis fellowship. Key factors influencing the decision to pursue a uveitis fellowship included the perceived complexity of the field (21.6%), job market perceptions (19.6%), and mentor influence (15.7%). Conversely, the main reasons for not choosing a uveitis fellowship were perceptions regarding surgical opportunities (17.6%), beliefs about the field’s complexity (15.1%), and salary expectations (13.2%). Residents not pursuing uveitis subspecialization were more likely to decide before their PGY3 year (26.7% vs. 14.3%, P = 0.041) and 43.6% indicated they would reconsider if the fellowship were combined with another subspecialty. Additionally, 75% (N = 87) felt their uveitis rotation time was inferior compared to other subspecialties. Residents pursuing uveitis subspecialization had more surgical exposure with uveitis-trained faculty than those who did not (64.3% vs. 56.4%, P = 0.018). Residents not pursuing a uveitis fellowship had a higher number of uveitis-trained faculty at their program compared to residents who pursued a fellowship in uveitis (1.90 vs. 1.14, P = 0.002).

**Conclusions:**

Improving recruitment into uveitis fellowship may require residency curriculum adjustments that emphasize earlier rotations and increased surgical exposure with uveitis faculty. While didactic and research opportunities are valuable, other factors may play a more determinative role in a resident’s decision to pursue a uveitis fellowship. Strengthening mentorship programs can address concerns compensation along with surgical and research opportunities to foster an interest in current residents.

## Introduction

Uveitis is a significant cause of visual impairment globally and causes 10-15% of the cases of blindness in the United States (1, 2) The incidence and prevalence of uveitis have been reported to be increasing over time, indicating the need for more specialists in this field (3, 4). Currently, there are only 178 uveitis specialists accepting new patients in the United States, and between 2018 and 2023, there was a 6% decrease in applicants matching into uveitis fellowship positions (5, 6). The gap between the need for uveitis specialists and the numbers of applicants pursuing uveitis fellowship training suggests that barriers exist which deter ophthalmology residents from choosing this subspecialty (6).

Previous studies have demonstrated that exposure to a specific specialty, mentorship, and perceived lifestyle significantly influence medical specialty selection (7). Given the importance of these factors, the role of these elements including exposure to uveitis during residency training through rotations, didactics, and procedures requires further investigation (8, 9). A recent survey of 72 current ophthalmology residents revealed that only 1.5% of respondents either have applied or will be applying for a uveitis fellowship (9). Given the pressing need for more specialists, we developed a survey to assess clinical, surgical, and research exposure to the subspecialty of uveitis. From the responses, we analyzed the factors affecting the decision to pursue a uveitis fellowship by ophthalmology residents in programs accredited by the Accreditation Council for Graduate Medical Education (ACGME) in the United States.

## Methods

### Study design

A cross-sectional survey was conducted between December 4, 2023 and July 4, 2024. The survey was adapted from a prior study on pursuing neuro-ophthalmology fellowship and consisted of 18 multiple choice questions divided into two sections (8). The study followed the tenets of the Declaration of Helsinki and was approved by the Johns Hopkins Hospital Institutional Review Board, IRB00402245.

### Outcome measures

The primary outcome measure was to identify the factors influencing ophthalmology residents’ decisions for either pursuing or forgoing uveitis as a subspecialty. Secondary outcome measures assessed the exposure to uveitis-focused research, didactics, and surgical procedures during training.

### Survey distribution

An online survey tool developed by the Association of University Professors of Ophthalmology (AUPO) was utilized to distribute the questionnaire to all residency program directors of ACGME-approved ophthalmology residencies in the United States between December 4, 2023, and February 20, 2024. To increase response rates, the survey was distributed a second time to all uveitis specialists via the American Uveitis Society listserv between May 14, 2024, and July 4, 2024. Uveitis specialists practicing within an academic department associated with a residency program were asked to distribute the survey to their residents. Given the use of two platforms for survey distribution, IP addresses of respondents were checked to exclude multiple responses.

### Statistical analysis

Two-sample independent t-tests were used to assess interval variables, chi-square test for ordinal variables, or the Fisher exact test for either categorical variables or dichotomous choices. Ordinal variables where >20% of expected cell counts contained 5 or fewer, the Fisher exact test was used. A two-sided P value of <0.05 was considered statistically significant. IBM SPSS Statistics for Windows, Version 29.0 was used for all analyses. The number of potential respondents was calculated from the San Francisco Match website from the residency matches from January 2021, 2022, and 2023.

## Results

**Figure 1:**
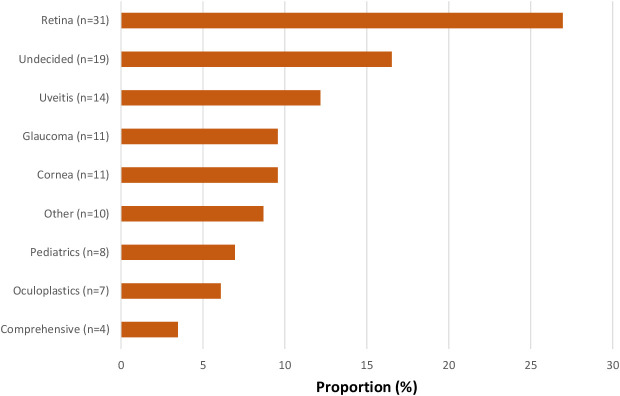
Distribution of specialty choice among residents.

A total of 115 responses were analyzed, including 28 from the AUPO distribution and 87 from the AUS listserv. Among these, 14 residents (12.2%) elected to pursue a uveitis fellowship. Utilizing SF Ophthalmology Match statistics from the past three years (N = 1,182), an approximate 9.7% response rate among current ophthalmology residents was obtained. The study has a margin of error of ±9% at a 95% confidence interval. **Figure 1** illustrates the distribution of specialty choices among the residents.

The majority of respondents (60%, N = 69) chose which specialty they wanted to pursue during PGY-2, regardless of which fellowship they ultimately pursued. Notably, a significantly higher proportion of residents who decided not to pursue a uveitis fellowship made their decision before PGY-3, whereas those who chose to specialize in uveitis made their decision more often during or after PGY-3(26.7% vs. 14.3%, respectively, P = 0.041). Additionally, 44 residents (43.6%) who chose not to pursue a uveitis fellowship indicated that they would reconsider if the fellowship was combined with another subspecialty.

**Table 1** presents the differences in perceived uveitis training and exposure between residents who chose or did not choose to pursue a uveitis fellowship. No significant differences were found in the exposure to the field of uveitis during medical school or in the number of residents who had a dedicated uveitis rotation. Furthermore, among those who had a uveitis rotation, there was no significant difference in the year they had the rotation, with PGY-3 being the most common in both groups. While 75% (N = 87) of residents felt that their uveitis rotation offered less time compared to other specialties, there was no significant difference between those who planned to or did not plan to subspecialize in uveitis. Similarly, there was no significant difference in the perceived quality of clinical exposure, with the majority in both groups reporting it to be on par with other specialties. However, those pursuing a uveitis fellowship were significantly more likely to report that the quality of didactics (42.9% vs. 10.9%, P = 0.002) and research exposure (57.1% vs. 34.7%, P = 0.025) was inferior to other specialties.

**Table 1:** Uveitis training exposure.

	Pursuing Uveitis (n=14)	Not pursuing Uveitis (n=101)	P-value
Had exposure to uveitis in medical school: N (%)	8 (57.1)	45 (44.6)	0.376 ^a^
Dedicated uveitis rotation in residency: N (%)	5 (35.7)	45 (44.6)	0.579 ^a^
Year of uveitis rotation in residency: N (%)			
PGY-1	2 (14.3)	2 (2.0)	0.061 ^b^
PGY-2	1 (7.1)	16 (15.8)	
PGY-3	3 (21.4)	38 (37.6)	
PGY-4	1 (7.1)	1 (1.0)	
Uveitis compared to other specialties:			
Time spent on rotation: N (%)			
Less	12 (85.7)	75 (74.3)	0.109 ^b^
Equal	1 (7.1)	15 (14.9)	
More	1 (7.1)	0 (0.0)	
Quality of clinical exposure: N (%)			
Inferior	6 (42.9)	28 (27.7)	0.211^b^
On par	4 (28.6)	52 (51.5)	
Superior	3 (21.4)	19 (18.8)	
Quality of didactics: N (%)			
Inferior	6 (42.9)	11 (10.9)	**0.002** ^b^
On par	5 (35.7)	62 (61.4)	
Superior	2 (14.3)	28 (27.7)	
Quality of research exposure: N (%)			
Inferior	8 (57.1)	35 (34.7)	**0.025** ^b^
On par	3 (21.4)	58 (57.4)	
Superior	2 (14.3)	7 (6.9)	
Timing of choosing subspecialty: N (%)			
Medical School	1 (7.1)	12 (11.9)	**0.041** ^b^
PGY-1	0	5 (5.0)	
PGY-2	1 (7.1)	10 (9.9)	
PGY-3	6 (42.9)	63 (62.4)	
PGY-4	4 (28.6)	10 (9.9)	

PGY, postgraduate year; a, chi-square test; b, Fisher exact test.

Results highlighted with bold text were statistically significant.

Residencies on average had between 1 and 2 (1.81; SD 0.88) uveitis-trained faculty. The group not pursuing uveitis had a significantly higher number of uveitis-trained faculty members compared to those pursuing uveitis subspecialization (1.90; SD 0.85 versus 1.14; SD 0.77, respectively, P = 0.002). Despite this, residents who chose to pursue a uveitis fellowship were significantly more likely to have had surgical exposure with uveitis-trained faculty compared to those who did not (64.3% versus 56.4%, respectively, P = 0.018). Cataracts, retina, and corneal surgeries were the most commonly performed procedures by uveitis-trained faculty. Characteristics of the uveitis-trained faculty are summarized in **Table 2**.

**Table 2:** Uveitis faculty characteristics.

	Pursuing Uveitis (n=14)	Not pursuing Uveitis (n=101)	P-value
Uveitis-trained faculty members: #	1.14 ± 0.77	1.90 ± 0.85	**0.002** ^c^
Uveitis faculty performed surgeries: N (%)	11 (78.6)	72 (71.3)	0.569^a^
Exposed to surgeries by uveitis specialist: N (%)	9 (64.3)	57 (56.4)	**0.018** ^a^
Type of surgery exposed to with uveitis specialist: N (%)			
Cataracts	6 (42.9%)	44 (43.6%)	0.960^a^
Retina	7 (50.0%)	32 (31.7%)	0.175^b^
Cornea	2 (14.3%)	6 (5.9%)	0.250^b^
Glaucoma	2 (14.3%)	0	0.028^b^
Oculoplastic	0	0	
Strabismus	0	4 (4.0%)	**0.591** ^a^

^a^ chi-square test.

^b^ Fisher exact test.

^c^ two-sample independent test.

Results highlighted with bold text were statistically significant.

**Figure 2:**
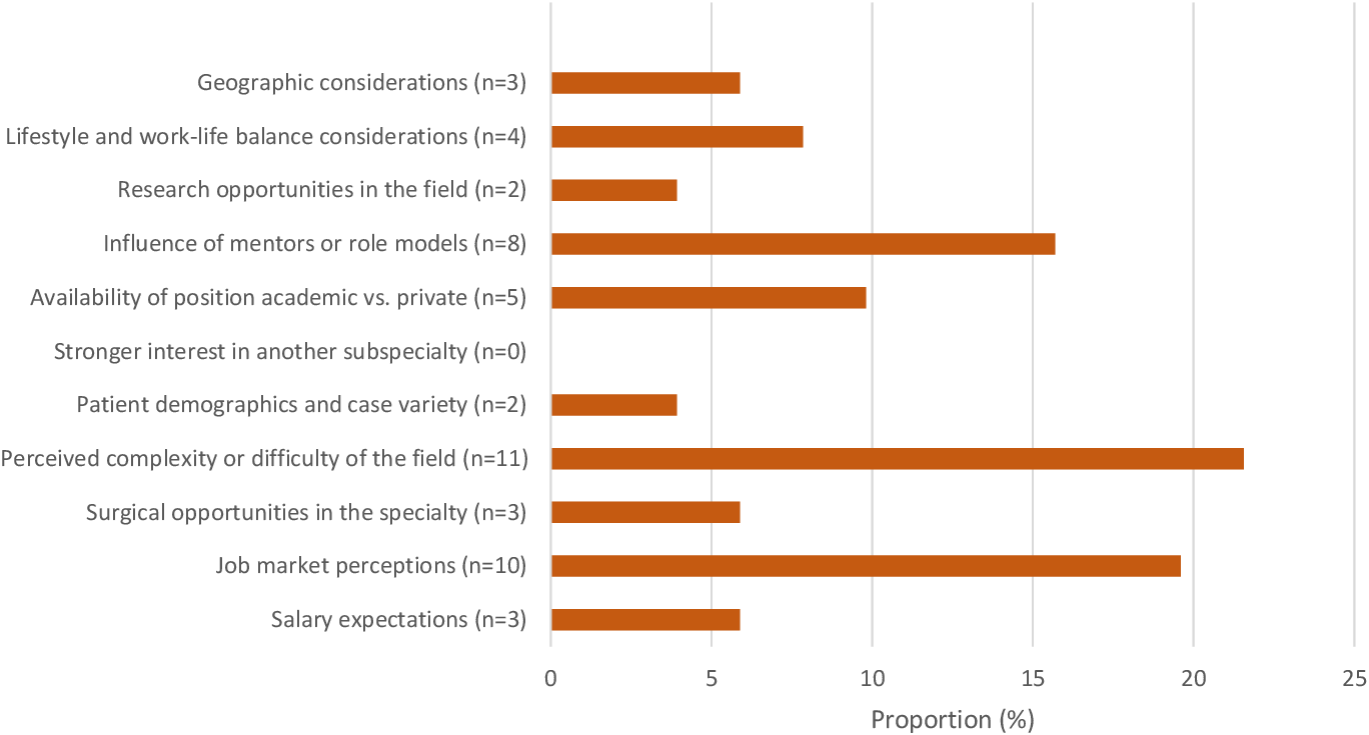
Factors influencing decision to pursue an uveitis fellowship.

**Figure 3:**
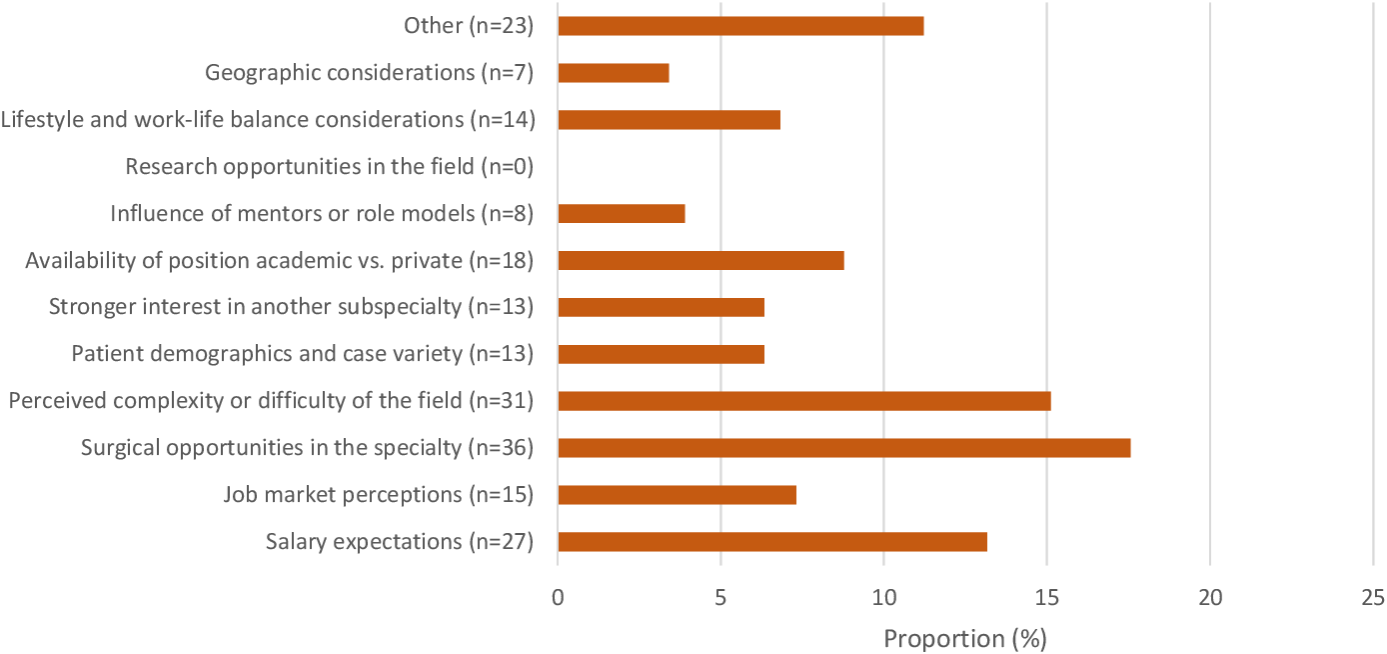
Factors influencing decision to not pursue an uveitis fellowship.

**Figures 2**, **3** present the factors influencing the decision to pursue or not pursue a uveitis fellowship. Among those pursuing a uveitis fellowship, the most common influencing factors were the complexity of the field (21.6%), perceptions of the job market (19.6%), and the influence of mentors (15.7%). Conversely, the most significant factors for those not pursuing uveitis subspecialization were perceived surgical opportunities (17.6%), perceived complexity (15.1%), and salary expectations (13.2%).

## Discussion

Although there has been a general increase in the proportion of ophthalmology residents applying to fellowship programs overall, this trend has not extended to the subspecialty of uveitis (9). This survey represents the first attempt to examine the attitudes and factors affecting the decision-making processes of ophthalmology residents on choosing a uveitis fellowship. Most of the surveyed residents (87.8%) chose not to pursue uveitis subspecialization, of these 43.6% stated they would reconsider their decision if the fellowship were combined with another subspecialty. This presents an opportunity in exploring the development of hybrid programs combining uveitis with another subspecialty, as an approach to improve recruitment, even if this may extend the duration of fellowship training. However, the survey also revealed other potential areas for improvement. Enhancing the quality of didactics and research exposure during residency rotations could foster greater interest in uveitis fellowships. Moreover, addressing residents’ perceptions about limited surgical opportunities and compensation could also increase the number of applicants.

One potential factor for recruitment is the timing and duration of uveitis rotations during residency. A majority of residents (60%) made their specialty choice during PGY-3, which was also the most common time for dedicated uveitis rotation. However, those who chose not to pursue uveitis fellowship training were more likely to have made their decision before the PGY-3 year. Also, the majority of respondents felt the time spent on a uveitis rotation was inferior to other subspecialties. Scheduling dedicated uveitis rotations early in the residency curriculum that have the equivalent number of weeks to other subspecialties may maximize residents obtaining necessary exposure to the field prior to making a fellowship choice.

Surgical exposure also differed between those who did and did not pursue uveitis fellowship training. Residents who pursued uveitis were significantly more likely to have been exposed to surgeries conducted by uveitis-trained specialists (64.3% vs. 56.4%, p=0.018). Notably, given the perception of limited surgical opportunities was the most commonly cited deterrent (17.6%) among those who chose another subspecialty. This suggests that misconceptions regarding surgical exposure due to lack of exposure may influence decision-making, highlighting the need for targeted efforts to improve awareness of surgical training opportunities within uveitis. Indeed, the heterogeneity of uveitis as a subspeciality with both medical and surgical practice patterns should be a potential advantage of the field. Efforts focusing on how a surgical practice can be tailored to an individual’s interest or combining uveitis with other surgical fellowships may be helpful in fostering interest amongst residents.

Despite uveitis having the highest mean papers per author and the highest H-index in a study comparing research productivity across ophthalmology subspecialties (10), residents pursuing a uveitis fellowship were more likely to perceive the quality of didactics and research opportunities in uveitis as inferior to other specialties. This perception may stem from factors such as the relatively smaller number of uveitis faculty in academic departments, which can limit the scope of uveitis lecture series for residents. To address this, developing research-focused mentorship programs and utilizing established virtual educational resources—such as those provided by the American Uveitis Society and Young Uveitis Society - may help foster interest and provide better support for residents interested in uveitis.

This study was limited by the method of distribution and timing of the survey. A relatively small portion of the current ophthalmology resident’s population responded, with 115 respondents representing approximately 9.7% of current residents; the margin of error is estimated to be ±9% at a 95% confidence interval. While this limits the generalizability of the results, given the exploratory nature of the study and the current lack of data regarding this subject, acceptable trends can still be identified for future research. Another limitation of this study is the lack of data regarding potential cofounding variables such as respondents’ socioeconomic status or fellowship availability in a desired area. Lastly, the percentage of residents that are pursuing uveitis is not representative of the general population given the bias introduced by the distribution method, largely through uveitis-trained faculty and residents interested in uveitis may be more likely to complete the survey.

Improving recruitment into uveitis fellowship may require residency curriculum adjustments that prioritize earlier uveitis rotations and increased surgical exposure with uveitis-trained faculty. Interestingly, our findings suggest that the perceived quality of uveitis didactics may not be a primary driver in the decision to pursue fellowship training. Residents who did not pursue uveitis were significantly more likely to rate their didactics as superior (27.7%), whereas those who chose to pursue uveitis were more likely to perceive them as subpar (42.9%; p=0.002). This suggests that while didactic strength is important, other factors—such as clinical and surgical exposure—may play a more determinative role in shaping a resident’s decision to specialize in uveitis. Given that mentors influence was a key determining factor for those pursing uveitis, strengthening mentorship programs and ensuring that residents have access to engaged and supportive uveitis-trained faculty may also make a difference in recruitment. Mentors can help navigate the job market and salary expectations through transparent discussions about the realities and opportunities in uveitis and may help in mitigating some of the misconceptions about a uveitis practice. Future studies may evaluate which aspects of mentorship, such as access to research and scholarly opportunities, influences interest in pursuing uveitis fellowship.

These findings indicate that a multi-faceted approach, including both structural changes to programs and improved education and mentorship during residency may be necessary to increase interest in uveitis as a subspecialty. Such changes could help narrow the gap between the growing need for uveitis specialists and the current shortage in the field.

## Data Availability

The raw data supporting the conclusions of this article will be made available by the authors, without undue reservation.
